# Challenges and potential of using digital biomarkers in healthcare and clinical trials

**DOI:** 10.1038/s43856-026-01450-8

**Published:** 2026-02-21

**Authors:** Johann K. Lieberwirth, Mirja Mittermaier, Ariel Dora Stern

**Affiliations:** 1 Hasso Plattner Institute, University of Potsdam, Potsdam, Germany; 2 Department of Infectious Diseases, Respiratory Medicine and Critical Care, Charité-Universitätsmedizin Berlin, Corporate Member of Freie Universität Berlin and Humboldt-Universität zu Berlin, Berlin, Germany; 3 Hasso Plattner Institute for Digital Health at Mount Sinai and Windreich Department of Artificial Intelligence & Human Health, Icahn School of Medicine at Mount Sinai, New York, NY USA

**Keywords:** Biomarkers, Health care, Biomarkers, Drug development, Clinical trial design

## Abstract

Digital biomarkers use sensors and analytics, to offer continuous monitoring and personalized medicine. In this Perspective, we describe real-world use cases, such as glucose tracking to optimise insulin dosing and wearables to measure heart rhythms to de-risk cardiovascular trials. We also discuss the issues preventing most candidate biomarkers from reaching clinical practice. Evidence generation is costly, regulatory reviews can be redundant, commercial incentives fall short, and data silos can be biased and/or nonrepresentative. By combining harmonized qualification pathways, value-based reimbursement, modular extensions for single-trial biomarkers, and adaptive post-market evidence loops, we propose a path from experimental signal to standard of care.

## Abstract (web summary)

Lieberwirth et al. discuss the current landscape of digital biomarkers in healthcare and clinical trials, highlighting growth in the use of digital endpoints and the emergence of dedicated “digital biomarker” startups. They discuss key barriers to translation and outline practical priorities for wider adoption.

Biomarkers are defined by the U.S. Food and Drug Administration (FDA) and the U.S. National Institutes of Health (NIH) as characteristics that can be measured to indicate normal biological processes, pathogenic processes, or responses to an exposure or intervention, including therapeutic interventions^1^. Traditional biomarkers, such as blood glucose levels or heart rate, have long been key to medical practice, serving as diagnostic, prognostic, or predictive tools^1,2^. In recent years, the integration of digital biomarkers in the healthcare delivery and clinical research settings has transformed both diagnostics and patient monitoring, enhancing the potential for early disease detection, personalized treatment, and continuous health monitoring.

Despite the increasing use of digital biomarkers, there is a notable lack of consensus on the definition of the term itself: a systematic review found that among 415 articles using the term “digital biomarker,” only 31% provided a clear definition, with 127 different definitions identified among those that did^3^. This lack of standardization is further complicated by the overlap between digital biomarkers and other health metrics, such as clinical outcome assessments, leading to confusion and conflation in their usage^4^. This variability complicates communication among researchers, clinicians, and regulators, highlighting the need for standardized terminology^1,4,5^.

For the purpose of this discussion, we adopt the definition provided by the European Medicines Agency (EMA): a digital biomarker is “an objective, quantifiable measure of physiology and/or behavior used as an indicator of biological, pathological process or response to an exposure or an intervention that is derived from a digital measure. The clinical meaning is established by a reliable relationship to an existing, validated endpoint”^6^. This definition underscores the requirement that the data collection, processing, and analysis must be inherently digital.

However, in some cases, this definition is not as clear as might be desired, as most biomarkers involve some degree of digital data processing in practice. We illustrate this by considering two examples, prostate-specific antigen (PSA) and continuous glucose monitoring (CGM).

PSA testing involves manual blood sample collection and biochemical assays, with digital technology applied only during data reporting. Therefore, PSA is not considered a digital biomarker under the EMA’s definition, as the measurement is not derived from a digital measure.

In contrast, CGM devices continuously monitor glucose levels through sensors that convert biochemical signals into digital data. Despite the initial biochemical interaction, CGM-derived glucose readings are considered digital biomarkers because the data collection, processing, and analysis are inherently digital. Integrating biochemical sensing with digital technology enables continuous monitoring and data-driven insights, aligning CGMs with the EMA’s definition of digital biomarkers.

The biomarkers, endpoints, and other tools (BEST) glossary developed by the FDA and NIH categorizes biomarkers into several subgroups based on their specific applications in healthcare and research, highlighting the broad spectrum of possible applications for biomarkers^1^. Table 1 presents examples of both traditional and digital biomarkers in each respective subgroup. A comprehensive version of this table, including definitions, can be found in Supplementary Data 1.

**Table 1:** Biomarker definitions and examples

Term	Classical/analog examples (taken from FDA BEST)^a^	Digital examples
Diagnostic biomarker	• HbA1c: for identifying and monitoring patients with diabetes mellitus • Glomerular filtration rate (GFR): for identifying patients with chronic kidney disease	• Digital ECG patterns: for diagnosing atrial fibrillation using wearable devices^56,57^ • Smartphone-based voice analysis: for early diagnosis of neurological conditions, such as Parkinson’s disease^58–61^
Monitoring biomarker	• HCV-RNA levels: for assessing treatment response in patients with chronic hepatitis C • CA-125: for monitoring the disease status in ovarian cancer treatment	• Remote monitoring: for heart rate or blood pressure in cardiac patients^62–64^ • Smart inhalers: for monitoring medication adherence^11,12^
Pharmacodynamic biomarker	• Circulating B lymphocytes: for assessing response to a B-lymphocyte stimulator inhibitor in systemic lupus erythematosus • INR (international normalized ratio): for evaluating response to warfarin treatment for thrombosis prevention	• Continuous heart rate monitoring: for assessing heart rate response to beta-blockers^65,66^ • Digital spirometry: for monitoring lung function in response to bronchodilator treatments in asthma patients^13,67,68^
Surrogate endpoint biomarker	• HbA1c reduction: for reducing microvascular complications in diabetes mellitus • HIV-RNA reduction: for controlling HIV clinical disease	• Continuous glucose monitoring: for predicting long-term outcomes in diabetes^16,69^ • AI-analyzed motor progression biomarkers: for tracking Parkinson’s disease progression using smartwatches to assess and quantify motor impairments^70–72^
Predictive biomarker	• Squamous differentiation in non-small cell lung cancer: for identifying patients who should avoid pemetrexed treatment • BRCA1/2 mutations: for predicting response to PARP inhibitors in platinum-sensitive ovarian cancer	• AI-predicted response to cancer immunotherapy based on: ○ radiography data^73^ ○ histology data^74^ ○ genetic data^75^
Prognostic biomarker	• Gene expression profiling: for segregating patients with diffuse large B-cell lymphoma into subgroups with different outcomes • Glomerular filtration rate (GFR): for predicting prognosis in chronic kidney disease	• Smartphone-based gait analysis for predicting fall risk in elderly patients using motion sensors and machine learning algorithms^76,77^ • Single-patient stroke outcome prediction^78–80^
Safety biomarker	• Liver enzymes (e.g., ALT, AST): for detecting liver toxicity from drugs • Serum creatinine: for assessing kidney damage or dysfunction due to drug toxicity	• Data from (portable) ECGs: for assessing risk of drug-induced Long-QT syndrome^81^
Susceptibility/risk biomarker	• Low-density lipoprotein (LDL) cholesterol: for evaluating the risk of cardiovascular disease • HPV DNA: for assessing risk of developing cervical cancer	• Heart rate variability: for assessing long-term cardiovascular risk^82–84^ • Sleep pattern monitoring via wearables: for assessing the risk of mental and chronic by analyzing sleep variability^85,86^

Examples for classical and digital biomarkers. Analog examples and definitions are taken from FDA BEST.

^a^A comprehensive version including definitions can be found in Supplementary Data 1.

## Clinical benefits and general opportunities

The European Union defines personalized medicine as “a medical model using characterization of individuals’ phenotypes and genotypes (e.g., molecular profiling, medical imaging, lifestyle data) for tailoring the right therapeutic strategy for the right person at the right time, and/or to determine the predisposition to disease and/or to deliver timely and targeted prevention”^7^. Biomarkers underpin this model by informing clinical decisions^8,9^. Digitally captured biomarkers amplify this concept: everyday devices gather continuous, non-invasive data, making large-scale, low-cost screening feasible and shifting detection to pre-symptomatic stages. Examples include fall-risk alerts derived from gait data on smartphones, sleep-apnea screening via wearables, and mobile ECGs to detect atrial fibrillation, where traditional screening is limited by cost constraints^10^.

Continuous monitoring is particularly promising for the management of chronic diseases. Wearable devices and smart inhalers can track respiratory rates, airflow, and oxygen saturation in COPD and asthma patients, detecting exacerbations before symptoms become severe and help monitor therapy adherence^11–14^. In diabetes, continuous glucose monitors provide real-time blood sugar trends for better insulin regulation^15,16^, while subtle changes in voice, body weight, or physical activity can indicate worsening heart failure, prompting timely intervention^17^. Indeed, conditions requiring precise dosage adjustments are better managed through real-world, ambulatory data rather than relying solely on intermittent clinic visits^15,18–20^.

For conditions with fluctuating severity, such as mental health disorders, passive monitoring of speech patterns, phone usage, and physical activity can detect early signs of mood changes, facilitating timely intervention^21^. This continuous, unobtrusive monitoring provides a more objective and nuanced understanding of symptom severity than traditional self-reporting.

Fields such as cardiology, endocrinology, neurology, and psychiatry are thus particularly poised to benefit from these innovations, as these represent settings in which continuous, objective ambulatory measurements can enable early detection, real-time monitoring, and personalized intervention. This is also reflected by the distribution of medical specialties represented by studies in the Digital Medicine Society’s *Library of Digital Endpoints* (DiMe *LDE*)^22^ (for more details, refer to the next section). By improving both the precision of diagnosis and the timeliness of care, digital biomarkers pave the way for a shift from reactive to proactive and preventive healthcare, ultimately enhancing patient outcomes.

## Opportunities in drug development and clinical trials

Biomarkers are utilized throughout the process of clinical development to reduce uncertainty and enhance the efficacy of decision-making. They have the capacity to facilitate enrichment and stratified analyses, provide early pharmacodynamic evidence of target engagement and dose response, monitor safety, and—in certain circumstances—act as surrogate endpoints when adequately validated for a defined context of use. In this section, the general trial functions are first outlined, and then the focus shifts to the extension of these functions using digital biomarkers, which enable remote, high-frequency measurements in real-world settings.

The cost of drug development is now estimated to exceed $1 billion per approved drug, with fewer than 1% of early-stage candidates reaching market approval^23,24^, and the number of FDA-approved drugs per billion U.S. dollars spent halving approximately every nine years^23^. Late-stage clinical trial failures have contributed to declining success rates, underscoring the urgency for more efficient trial methodologies. Insufficient efficacy is the predominant reason for candidate failure, highlighting the need for both improved patient stratification and trial design^25^.

Biomarker-driven assessments in Pfizer’s clinical trials increased phase II success rates from 19 to 53%, substantially reducing costly late-stage failures^26^. By enabling early proof-of-mechanism studies, optimizing dosing, and improving patient stratification, biomarkers helped limit the number of ineffective compounds advancing to phase III. This experience highlights the growing importance of biomarker integration in clinical trial design^26^.

Biomarkers serve as critical tools in optimizing clinical trials and, therefore, new therapeutic development by refining patient selection and increasing the probability of therapeutic success^8^. Stratification based on biomarkers enhances the detection of clinically meaningful effects in specific subpopulations. Previous studies have shown that biomarkers—and, increasingly, digital endpoints—can significantly reduce cohort sizes, shorten study duration, and increase success rates^27–29^. These efficiency gains directly translate to reduced costs and accelerated timelines for drug approval^30^.

Beyond improving efficiency, biomarkers provide essential mechanistic insights that inform drug development strategies. Retrospective analyses enable the identification of responsive subpopulations, justifying continued investment in promising compounds^8^. Although this approach necessitates additional upfront costs for sample collection and storage, it can potentially salvage projects that might otherwise be terminated due to apparent inefficacy.

Even when clinical trials fail, biomarkers can provide actionable insights. If a biomarker confirms full target engagement but no therapeutic benefit is observed, this suggests that the drug’s molecular target is not a key driver of the disease^8,26^. Conversely, insufficient target engagement may indicate inadequate drug potency, bioavailability, or dosing, rather than a fundamental failure of the therapeutic approach. These insights refine future drug development strategies, benefiting not only the immediate stakeholders but also the broader biomedical research and development community^8,31^.

Digital biomarkers represent a subset of biomarkers derived from digital measures and digital health technologies, allowing continuous or frequent remote data capture that can support decentralized trial designs and potentially improve efficiency and participant experience. A study analyzing trials from the DiMe *LDE* found that Phase III trials incorporating digital biomarkers were, on average, 4–5 months shorter and had 11.7% fewer participants. The average return on investment of initial implementation costs of digital endpoints ranged between 31 and 48% for phase II trials and 350 and 590% for phase III trials^32^.

The scale of potential efficiency gains is striking^33^. Holding 80% power, a 30% effect size, and a four-year horizon constant, conventional memory testing required an estimated 2000 participants; a digital gait outcome reduced the need to 262, and a computer-use endpoint to 26. By capturing high-frequency behavior in naturalistic, real-world settings, digital biomarkers can deliver orders-of-magnitude reductions in sample size, with direct implications for timelines and cost.

Digital biomarkers are revolutionizing decentralized clinical trials (DCTs) by minimizing dependence on centralized research facilities and leveraging home-based data collection through wearables and telemedicine^34^. These typically passive measurements demand little to no effort from patients, significantly enhancing adherence, particularly among populations with historically lower compliance^35,36^. Unlike traditional trials, which are reliant on sporadic clinic visits, DCTs enable continuous or frequent data collection, yielding richer datasets and more reliable insights with fewer clinic visits. Beyond logistical advantages, DCTs improve trial efficiency on multiple fronts. They facilitate faster participant recruitment, bolster retention rates, and enhance patient autonomy and convenience. Additionally, by lowering enrollment barriers, DCTs contribute to greater trial diversity, ensuring outcomes that are more representative of real-world populations^36^.

**Fig. 1:**
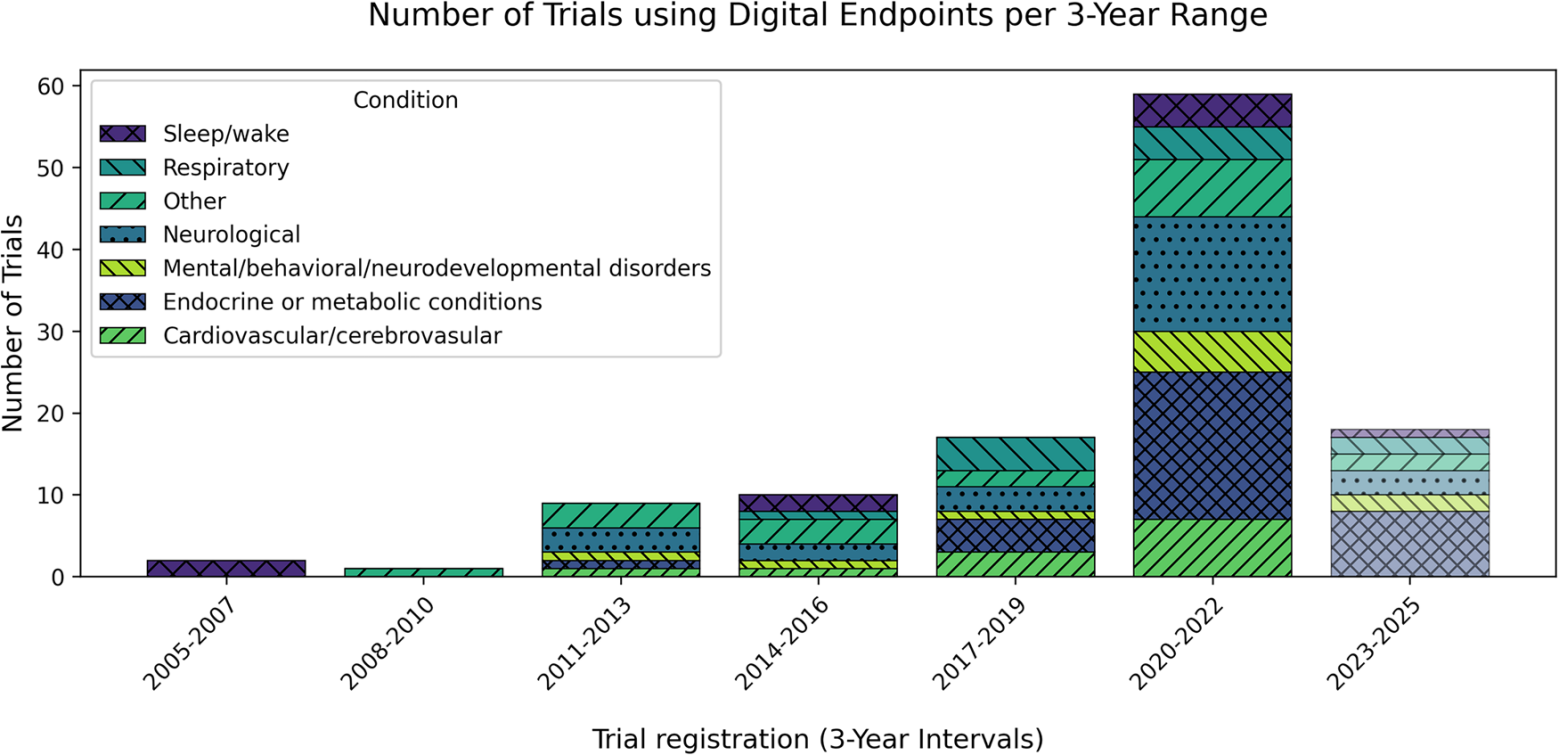
Clinical trials using digital endpoints over time, by condition category (2005–2024) Number of trials using digital endpoints, grouped into 3-year bins from 2005 to 2024^22^. The top six condition categories are shown, with the remainder classified as “other.” The final bin (2023–2025) is semi-transparent to indicate that the data are preliminary.

At the time of analysis, the DiMe *LDE* cataloged 102 clinical trials employing digital endpoints, with 44 using them as primary measures and 79 incorporating them as secondary endpoints^22^. The majority of these trials focus on endocrine/metabolic (30.4%) and neurological conditions (24.5%), followed by cardiovascular, respiratory, and mental health disorders (Fig. 1). The increasing prevalence of digital biomarkers in clinical trials reflects improved feasibility of remote, high-frequency data capture, the advancement of digital health technologies in recent years, and the practical appeal of more convenient data collection approaches.

## Challenges and regulatory considerations

The path from digital biomarker discovery to clinical use is marked by significant translational barriers. Most biomarkers do not advance from research settings to regulatory approval and broad clinical implementation, in part owing to complex validation protocols, stringent regulatory requirements, and integration challenges within healthcare infrastructure^37–39^. Regulatory pathways, particularly “biomarker qualification programs” from regulators, such as the FDA and the EMA, that enable broad use across different contexts of use, remain complex and slow, with only a few biomarkers successfully qualified to date^40,41^. Consequently, many biomarkers are confined to single-trial validation, limiting their broader impact.

These system-level obstacles are compounded by measurement-specific issues. Ensuring that digital biomarkers are both methodologically robust and clinically meaningful requires adherence to rigorous evidence-generation frameworks, such as the Verification-Analytical Validation-Clinical Validation (V3) paradigm^5^. Unlike many traditional biomarkers, such as genetic mutations and blood tests, that have direct mechanistic links to disease pathology and well-established reference standards, digital biomarkers are derived from sensors and algorithms and often must be interpreted within a defined clinical context. The V3 framework structures this work into three linked questions:

- *Verification*: do sensors and devices meet predefined technical specifications and reliably capture the intended raw signal? Example: bench testing an accelerometer-based system to confirm accurate motion capture under controlled conditions.
- *Analytical validation*: do the algorithms transform raw signals into accurate, reproducible measures? Example: comparing algorithm-derived step counts with a gold-standard manual tally and assessing agreement, repeatability, and drift.
- *Clinical validation*: does the resulting measure answer a specific clinical question in its context of use? Example: testing whether step-count or gait metrics predict mobility improvement in patients undergoing rehabilitation or track clinically meaningful change over time.

Additional challenges arise from symptom variability across patients. In diseases such as Parkinson’s, Alzheimer’s, or depression, symptoms manifest differently and fluctuate over time. A biomarker that reliably detects tremor in one subgroup of Parkinson’s patients may not apply to those whose dominant symptoms are rigidity or bradykinesia. As a result, analyses may need to be stratified by phenotype or symptom domain to avoid misleading conclusions. Day-to-day variation related to medication cycles, stress, or fatigue further complicates validation and reproducibility.

A further issue is the absence of gold standards for many digital biomarkers^38^. Unlike traditional biomarkers with well-established reference measures, such as red blood cell counts in anemia, digital biomarkers often lack direct clinical equivalents. For example, a tool that detects vocal markers of depression must be compared to clinical assessments rather than objective biological markers, complicating accuracy targets and thresholds. Context dependence adds another layer: digital biomarkers may capture proxy variables rather than the condition itself. A wearable detecting reduced hand movement may suggest Parkinson’s, but could also indicate arthritis or fatigue.

Even when a digital measure detects a pattern, it must be clinically validated against outcomes that matter to patients. The FDA’s initial rejection of the Verily study watch for Parkinson’s illustrates this challenge: despite alignment with the Movement Disorder Society-Unified Parkinson’s disease rating scale (MDS-UPDRS), it was deemed insufficient for capturing meaningful patient function. These difficulties are especially pronounced in neurodegenerative and psychiatric diseases, where symptom expression is heterogeneous and hard to standardize. To mitigate this, a framework has been proposed that anchors digital measures to patient-relevant outcomes, improving the likelihood of regulatory and clinical acceptance^42^.

### Practical considerations in population screening

Beyond therapeutic applications, digital biomarkers are increasingly deployed for early disease detection and broad population screening. As with every test, false positives can lead to unnecessary follow-up tests and treatments, while false negatives risk preventing individuals who need timely intervention from seeking professional evaluation. Even when these tests perform well, they can introduce new challenges: there is a growing number of asymptomatic individuals, often significantly younger than the average cardiac patient, alerted by their smartwatches about intermittent atrial fibrillation. For this population, the benefits of anticoagulant therapies remain unclear, raising concerns about how to appropriately address these findings in practice and their broader implications for public health^10,43,44^.

### Algorithmic bias and healthcare inequities

Further ethical concerns emerge regarding algorithmic bias and disparities in digital health access. Surveys indicate that individuals with higher income, higher education levels, and those residing in urban areas are more likely to own and use wearable devices^45^. Developing digital health solutions primarily for these platforms without addressing barriers to access may exacerbate existing health inequities. Algorithmic bias in data processing and interpretation can disproportionately affect minority groups, necessitating rigorous fairness assessments in biomarker validation^46^. A prominent example of this kind of bias is the underrepresentation of diverse skin colors in datasets used to train skin cancer detection algorithms, which can lead to biased predictions for underrepresented groups^47^.

### Misaligned financial incentives

Beyond these scientific and clinical challenges, financial incentives and regulatory hurdles further shape biomarker development^48^. While biomarkers can enhance drug development and precision medicine, industry investment is driven by market dynamics, such as pricing strategies and competitive advantages^49,50^. Predictive biomarkers inherently narrow a drug’s eligible patient pool, which threatens sales volumes, however, current reimbursement frameworks rarely allow higher prices to offset this loss, so manufacturers gain little financial return on the substantial R&D cost of biomarker development. Explicit pricing rewards or coverage incentives for precision-guided therapies, motivates drug companies to invest in validating and commercializing predictive biomarker tests^49^.

## Future directions, conclusions and recommendations

Looking ahead, advances in digital phenotyping and the use of patient “digital twins” could further support personalized healthcare. Digital phenotyping allows for the continuous, high-resolution monitoring of individual health trajectories through everyday digital interactions^51^. Digital patient twins, virtual representations of an individual that are continuously updated with longitudinal, multi-source patient data (e.g., EHR, imaging, and wearable/IoT streams), coupled with computational models (often incorporating AI/ML), promise to refine treatment simulations and optimize intervention strategies^52^. Their use will almost certainly increase as U.S. policy encourages pharmaceutical R&D to move away from the use of animal models in drug development^53^. These technologies have far-reaching implications beyond clinical settings, extending to public health surveillance, occupational health, and actuarial modeling in the insurance sector.

**Fig. 2:**
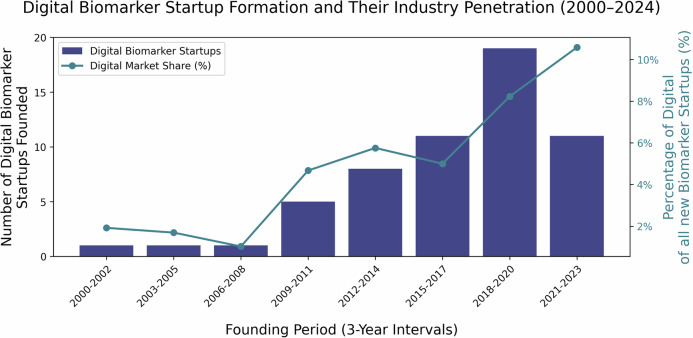
Digital biomarker startup formation and share among biomarker companies (PitchBook, 2000–2024) Number of companies that mention the term “digital biomarker” in either the PitchBook^54^ “description” or “keywords” fields, founded within 3-year intervals from 2000 to 2024. A total of 57 such companies are included in this analysis. The bars (in blue) show the absolute count of digital biomarker startups for each interval. Overlaid is a line plot (in green) indicating the percentage of digital biomarker startups among all biomarker companies founded in the same period. The left y-axis displays the count of digital biomarker startups, while the right y-axis shows the digital share of newly founded biomarker startups as a percentage.

The entrepreneurial landscape reflects this rapid development, with an increasing number of startups focusing on digital biomarkers (Fig. 2). An analysis of venture-funded companies listed in PitchBook reveals that investment in this sector has surged, particularly between 2018 and 2021, driven by advances in wearable technology, data analytics, and personalized medicine^54^. Although there has been a post-2021 funding slowdown due to broader market corrections, the continued interest in digital health solutions highlights the commercial potential of digital biomarkers.

Despite their potential, significant challenges remain. Validation and standardization efforts are critical, as digital biomarkers often lack universally accepted benchmarks compared to traditional biomarkers. Regulatory uncertainty, ethical concerns related to data privacy and algorithmic bias, and potential healthcare inequities must be addressed to ensure fair and effective implementation.

In order to overcome the persistent barriers that currently exist, coordinated action is likely to be needed. In the immediate future, there are several pragmatic measures that must be taken by sponsors, investigators, and developers:

- *Adopt regulator-aligned terminology and ontology*: explicitly map key terms to the FDA BEST glossary and relevant EMA guidance in manuscripts and protocols (e.g., a short definitions table), avoiding trial-specific redefinitions.
- *Embed fit-for-purpose validation from the outset*: digital-biomarker programmes should adopt the V3 framework, a step-wise process that first confirms reliable signal capture, then demonstrates assay accuracy and reproducibility, and finally proves real-world clinical relevance^5^. Implementing V3 at the concept stage ensures that algorithms, assay precision and patient benefit are established before large-scale deployment, thereby meeting the evidentiary thresholds that currently impede regulatory progress. Frameworks can help align digital endpoints with clinically meaningful outcomes, ensuring that validation demonstrates both clinical effect and patient-relevant benefit, thereby meeting regulatory requirements^42^.
- *Plan for re-use via comparability evidence*: when extending a single-trial biomarker to new contexts of use, maintain measurement consistency (sensor mechanism, device configuration, algorithm/software version) where feasible; when technology changes, pre-specify bridging/comparability testing to demonstrate analytical equivalence.
- *Institutionalize equity and bias safeguards*: pre-register bias audits (including subgroup performance), document training/validation dataset representativeness, and include an access plan (e.g., device provision/loan models) when endpoints rely on consumer hardware.

Longer-term needs (regulators/payers/health systems)

- *More predictable regulatory pathways*: expand and clarify qualification/acceptance routes for digital endpoints and define expectations for cross-trial re-use (including comparability requirements).
- *Reduce duplicative review across agencies*: explore mechanisms for regulatory reliance, shared scientific advice, or aligned evidentiary packages across major regulators to lower transaction costs and timelines. One example of an efficiency-driving approach can be seen in the guiding principles for “predetermined change control plans for machine learning-enabled medical devices” jointly published by the FDA, Health Canada, and the U.K.’s Medicines and Healthcare Products Regulatory Agency (MHRA)^55^.
- *Align incentives with demonstrated value*: develop reimbursement and coverage approaches that reflect incremental clinical value when biomarker-guided strategies improve outcomes, efficiency, or patient experience.

Taken together, these near-term steps and longer-term system needs can strengthen the evidence base and implementation pathway for digital biomarkers. Digital biomarkers have the potential to contribute to more efficient clinical trials, accellerate the development of new therapeutics, and, in selected contexts, more individualized care. This is predicated on rigorous fit-for-purpose validation, clearer regulatory expectations, and equity- and privacy-aware governance. The following priorities have been identified for the forthcoming years: targeted methodological research, improved regulatory convergence, and transparent, ethically grounded deployment.

## Supplementary information

Description of Additional Supplementary Files

Supplementary Data 1

## References

[1] FDA-NIH Biomarker Working Group. *BEST* (*Biomarkers, EndpointS, and Other Tools*) (FDA-NIH, 2021).

[2] Babrak, L. M. et al. Traditional and digital biomarkers: two worlds apart? *Digit. Biomark.* **3**, 92–102 (2019). 32095769 10.1159/000502000 PMC7015353

[3] Alonso, A. K. M., Hirt, J., Woelfle, T., Janiaud, P. & Hemkens, L. G. Definitions of digital biomarkers: a systematic mapping of the biomedical literature. *BMJ Health Care Inform.* **31**, e100914 (2024). 10.1136/bmjhci-2023-100914 PMC11015196 38589213

[4] Vasudevan, S., Saha, A., Tarver, M. E. & Patel, B. Digital biomarkers: convergence of digital health technologies and biomarkers. *NPJ Digit. Med.* **5**, 1–3 (2022). 35338234 10.1038/s41746-022-00583-z PMC8956713

[5] Goldsack, J. C. et al. Verification, analytical validation, and clinical validation (V3): the foundation of determining fit-for-purpose for biometric monitoring technologies (BioMeTs). *NPJ Digit. Med.* **3**, 1–15 (2020). 32337371 10.1038/s41746-020-0260-4 PMC7156507

[6] European Medicines Agency. Questions and answers: Qualification of digital technology-based methodologies to support approval of medicinal products. EMA/219860/2020 (European Medicines Agency, 2020). Accessed 11 Feb 2025. https://www.ema.europa.eu/en/documents/other/questions-and-answers-qualification-digital-technology-based-methodologies-support-approval-medicinal-products_en.pdf

[7] *Council Conclusions on Personalized Medicine for Patients* https://eur-lex.europa.eu/legal-content/EN/TXT/PDF/?uri=OJ:C:2015:421:FULL (2015).

[8] Landeck, L., Kneip, C., Reischl, J. & Asadullah, K. Biomarkers and personalized medicine: current status and further perspectives with special focus on dermatology. *Exp. Dermatol.* **25**, 333–339 (2016). 27167702 10.1111/exd.12948

[9] Meister, S., Deiters, W. & Becker, S. Digital health and digital biomarkers–enabling value chains on health data. *Curr. Dir. Biomed. Eng.* **2**, 577–581 (2016).

[10] Mandrola, J. & Foy, A. Downsides of detecting atrial fibrillation in asymptomatic patients. *Am. Fam. Physician* **99**, 354–355 (2019). 30874403

[11] Burgess, S. W., Wilson, S. S. I., Cooper, D. M., Sly, P. D. & Devadason, S. G. In vitro evaluation of an asthma dosing device: the smart-inhaler. *Respir. Med.* **100**, 841–845 (2006). 16216485 10.1016/j.rmed.2005.09.004

[12] Jansen, E. M. et al. Global burden of medication non-adherence in chronic obstructive pulmonary disease (COPD) and asthma: a narrative review of the clinical and economic case for smart inhalers. *J. Thorac. Dis.* **13**, 3846 (2021). 34277075 10.21037/jtd-20-2360 PMC8264677

[13] Gupta, S., Chang, P., Anyigbo, N. & Sabharwal, A. MobileSpiro: accurate mobile spirometry for self-management of asthma. In *Proc. First ACM Workshop on Mobile Systems, Applications, and Services for Healthcare* 1–6 (Association for Computing Machinery, 2011).

[14] Nathan, V., Vatanparvar, K., Rahman, M. M., Nemati, E. & Kuang, J. Assessment of chronic pulmonary disease patients using biomarkers from natural speech recorded by mobile devices. In *Proc. 2019 IEEE 16th International Conference on Wearable and Implantable Body Sensor Networks (BSN)* 1–4 (IEEE, 2019).

[15] Rodbard, D. Continuous glucose monitoring: a review of successes, challenges, and opportunities. *Diabetes Technol. Ther.* **18**, S2–S3 (2016). 26784127 10.1089/dia.2015.0417 PMC4717493

[16] Battelino, T. et al. Continuous glucose monitoring and metrics for clinical trials: an international consensus statement. *Lancet Diabetes Endocrinol.* **11**, 42–57 (2023). 36493795 10.1016/S2213-8587(22)00319-9

[17] Maor, E. et al. Vocal biomarker is associated with hospitalization and mortality among heart failure patients. *J. Am. Heart Assoc.* **9**, e013359 (2020). 32233754 10.1161/JAHA.119.013359 PMC7428646

[18] Truong, A. T. L. et al. CURATE.AI-assisted dose titration for anti-hypertensive personalized therapy: study protocol for a multi-arm, randomized, pilot feasibility trial using CURATE.AI (CURATE.AI ADAPT trial). *Eur. Heart J. Digit. Health* **5**, 41–49 (2024). 38264697 10.1093/ehjdh/ztad063 PMC10802822

[19] Amir, O. et al. Evaluation of remote dielectric sensing (ReDS) technology-guided therapy for decreasing heart failure re-hospitalizations. *Int. J. Cardiol.* **240**, 279–284 (2017). 28341372 10.1016/j.ijcard.2017.02.120

[20] Wong, C. K. et al. Daily ambulatory remote monitoring system for drug escalation in chronic heart failure with reduced ejection fraction: pilot phase of DAVID-HF study. *Eur. Heart J. Digit. Health* **3**, 284–295 (2022). 36713022 10.1093/ehjdh/ztac024 PMC9708020

[21] Low, D. M., Bentley, K. H. & Ghosh, S. S. Automated assessment of psychiatric disorders using speech: a systematic review. *Laryngoscope Investig. Otolaryngol.* **5**, 96–116 (2020). 32128436 10.1002/lio2.354 PMC7042657

[22] Digital Medicine Society (DiMe) Library of Digital Endpoints. *Digital Medicine Society**(DiMe)* https://dimesociety.org/communication-education/library-of-digital-endpoints/.

[23] Scannell, J. W., Blanckley, A., Boldon, H. & Warrington, B. Diagnosing the decline in pharmaceutical R&D efficiency. *Nat. Rev. Drug Discov.* **11**, 191–200 (2012). 22378269 10.1038/nrd3681

[24] Wouters, O. J., McKee, M. & Luyten, J. Estimated research and development investment needed to bring a new medicine to market, 2009-2018. *JAMA* **323**, 844–853 (2020). 32125404 10.1001/jama.2020.1166 PMC7054832

[25] Harrison, R. K. Phase II and phase III failures: 2013–2015. *Nat. Rev. Drug Discov.* **15**, 817–818 (2016). 27811931 10.1038/nrd.2016.184

[26] Wu, S. S. et al. Reviving an R&D pipeline: a step change in the phase II success rate. *Drug Discov. Today* **26**, 308–314 (2021). 33129994 10.1016/j.drudis.2020.10.019

[27] Servais, L. et al. First regulatory qualification of a novel digital endpoint in Duchenne muscular dystrophy: a multi-stakeholder perspective on the impact for patients and for drug development in neuromuscular diseases. *Digit. Biomark.* **5**, 183–190 (2021). 34723071 10.1159/000517411 PMC8460979

[28] Mori, H., Wiklund, S. J. & Zhang, J. Y. Quantifying the benefits of digital biomarkers and technology-based study endpoints in clinical trials: Project Moneyball. *Digit. Biomark.* **6**, 36–46 (2022). 35949224 10.1159/000525255 PMC9297703

[29] Chen, C. et al. Wrist-worn sensor-based measurements for drug effect detection with small samples in people with Lewy body dementia. *Park. Relat. Disord.* **109**, 105355 (2023). 10.1016/j.parkreldis.2023.105355 36905719

[30] Leptak, C. et al. What evidence do we need for biomarker qualification? *Sci. Transl. Med.* **9**, eaal4599 (2017). 29167393 10.1126/scitranslmed.aal4599

[31] Krieger, J. L. Trials and terminations: learning from competitors’ R&D failures. *Manag. Sci.* **67**, 5525–5548 (2021).

[32] DiMasi, J. A. et al. Assessing the net financial benefits of employing digital endpoints in clinical trials. *Clin. Transl. Sci.* **17**, e13902 (2024). 39072949 10.1111/cts.13902 PMC11284240

[33] Dodge, H. H. et al. Use of high-frequency in-home monitoring data may reduce sample sizes needed in clinical trials. *PLoS ONE* **10**, e0138095 (2015). 26379170 10.1371/journal.pone.0138095 PMC4574479

[34] Mittermaier, M., Venkatesh, K. P. & Kvedar, J. C. Digital health technology in clinical trials. *NPJ Digit. Med.* **6**, 88 (2023). 37202443 10.1038/s41746-023-00841-8 PMC10195788

[35] Insel, T. R. Digital phenotyping: technology for a new science of behavior. *JAMA* **318**, 1215 (2017). 28973224 10.1001/jama.2017.11295

[36] Coravos, A. et al. Digital medicine: a primer on measurement. *Digit. Biomark.* **3**, 31–71 (2019). 32095767 10.1159/000500413 PMC7015383

[37] Poste, G. Bring on the biomarkers. *Nature* **469**, 156–157 (2011). 21228852 10.1038/469156a

[38] Lavezzari, G. & Womack, A. W. Industry perspectives on biomarker qualification. *Clin. Pharmacol. Ther.* **99**, 208–213 (2016). 26378777 10.1002/cpt.264 PMC5065241

[39] U.S. Food and Drug Administration, Center for Drug Evaluation and Research (CDER) & Center for Biologics Evaluation and Research (CBER). Qualification Process for Drug Development Tools: Guidance for Industry and FDA Staff. (FDA, Nov 2020). Accessed 11 Feb 2025. https://www.fda.gov/media/133511/download

[40] U.S. Food and Drug Administration, Center for Drug Evaluation and Research (CDER), CDER COA Qualification Program (CDER COAQP). COAQP Information Session. (FDA, n.d.). Accessed 11 Feb 2025. https://www.fda.gov/media/151216/download

[41] Shahzad, M. & Stern, A. D. Participants in the FDA’s biomarker qualification program. *Clin. Pharmacol. Ther*. 10.1002/cpt.3661 (2025). 10.1002/cpt.3661 40202162

[42] Manta, C., Patrick-Lake, B. & Goldsack, J. C. Digital measures that matter to patients: a framework to guide the selection and development of digital measures of health. *Digit. Biomark.* **4**, 69–77 (2020). 33083687 10.1159/000509725 PMC7548919

[43] Shah, S. J., Eckman, M. H., Aspberg, S., Go, A. S. & Singer, D. E. Effect of variation in published stroke rates on the net clinical benefit of anticoagulation for atrial fibrillation. *Ann. Intern. Med.* **169**, 517–527 (2018). 30264130 10.7326/M17-2762

[44] Gibson, C. M. et al. Does early detection of atrial fibrillation reduce the risk of thromboembolic events? Rationale and design of the Heartline study. *Am. Heart J.* **259**, 30–41 (2023). 36642226 10.1016/j.ahj.2023.01.004

[45] Nagappan, A., Krasniansky, A. & Knowles, M. Patterns of ownership and usage of wearable devices in the United States, 2020-2022: survey study. *J. Med. Internet Res.* **26**, e56504 (2024). 39058548 10.2196/56504 PMC11316147

[46] Obermeyer, Z., Powers, B., Vogeli, C. & Mullainathan, S. Dissecting racial bias in an algorithm used to manage the health of populations. *Science* **366**, 447–453 (2019). 31649194 10.1126/science.aax2342

[47] Guo, L. N., Lee, M. S., Kassamali, B., Mita, C. & Nambudiri, V. E. Bias in, bias out: underreporting and underrepresentation of diverse skin types in machine learning research for skin cancer detection—a scoping review. *J. Am. Acad. Dermatol.* **87**, 157–159 (2022). 34252465 10.1016/j.jaad.2021.06.884

[48] Stern, A. D., Alexander, B. M. & Chandra, A. Innovation incentives and biomarkers. *Clin. Pharmacol. Ther.* **103**, 34–36 (2018). 29034452 10.1002/cpt.876

[49] Brekke, K. R., Dalen, D. M. & Straume, O. R. Competing with precision: incentives for developing predictive biomarker tests. *Scand. J. Econ.* **126**, 60–97 (2024).

[50] Antoñanzas, F., Juárez-Castelló, C. A. & Rodríguez-Ibeas, R. Pre-approval incentives to promote adoption of personalized medicine: a theoretical approach. *Health Econ. Rev.* **9**, 28 (2019). 31664604 10.1186/s13561-019-0244-8 PMC6820936

[51] Torous, J., Kiang, M. V., Lorme, J. & Onnela, J.-P. New tools for new research in psychiatry: a scalable and customizable platform to empower data driven smartphone research. *JMIR Ment. Health* **3**, e5165 (2016). 10.2196/mental.5165 PMC4873624 27150677

[52] Coorey, G. et al. The health digital twin to tackle cardiovascular disease—a review of an emerging interdisciplinary field. *NPJ Digit. Med.* **5**, 1–12 (2022). 36028526 10.1038/s41746-022-00640-7 PMC9418270

[53] FDA. FDA Announces Plan to Phase Out Animal Testing Requirement for Monoclonal Antibodies and Other Drugs. *FDA* https://www.fda.gov/news-events/press-announcements/fda-announces-plan-phase-out-animal-testing-requirement-monoclonal-antibodies-and-other-drugs (2025).

[54] PitchBook, D. PitchBook. *Venture Capital*, *Private Equity and M&A Database* https://pitchbook.com/.

[55] FDA. Predetermined Change Control Plans for Machine Learning-Enabled Medical Devices: Guiding Principles. https://www.fda.gov/medical-devices/software-medical-device-samd/predetermined-change-control-plans-machine-learning-enabled-medical-devices-guiding-principles (2025).

[56] Tison, G. H. et al. Passive detection of atrial fibrillation using a commercially available smartwatch. *JAMA Cardiol* **3**, 409–416 (2018). 29562087 10.1001/jamacardio.2018.0136 PMC5875390

[57] Perez, M. V. et al. Large-scale assessment of a smartwatch to identify atrial fibrillation. *N. Engl. J. Med.* **381**, 1909–1917 (2019). 31722151 10.1056/NEJMoa1901183 PMC8112605

[58] Rusz, J. et al. Smartphone allows capture of speech abnormalities associated with high risk of developing Parkinson’s disease. *IEEE Trans. Neural Syst. Rehabil. Eng.* **26**, 1495–1507 (2018). 29994713 10.1109/TNSRE.2018.2851787

[59] Wroge, T. J. et al. Parkinson’s disease diagnosis using machine learning and voice. In *Proc. 2018 IEEE Signal Processing in Medicine and Biology Symposium (SPMB)* 1–7 (IEEE, 2018).

[60] Karaman, O., Çakın, H., Alhudhaif, A. & Polat, K. Robust automated Parkinson disease detection based on voice signals with transfer learning. *Expert Syst. Appl.* **178**, 115013 (2021).

[61] Laganas, C. et al. Parkinson’s disease detection based on running speech data from phone calls. *IEEE Trans. Biomed. Eng.* **69**, 1573–1584 (2022). 34596531 10.1109/TBME.2021.3116935

[62] Georgiou, K. et al. Can wearable devices accurately measure heart rate variability? A systematic review. *Folia Med.* **60**, 7–20 (2018). 10.2478/folmed-2018-0012 29668452

[63] Sana, F. et al. Wearable devices for ambulatory cardiac monitoring. *J. Am. Coll. Cardiol.* **75**, 1582–1592 (2020). 32241375 10.1016/j.jacc.2020.01.046 PMC7316129

[64] Kuwabara, M., Harada, K., Hishiki, Y. & Kario, K. Validation of two watch-type wearable blood pressure monitors according to the ANSI/AAMI/ISO81060-2:2013 guidelines: Omron HEM-6410T-ZM and HEM-6410T-ZL. *J. Clin. Hypertens.* **21**, 853–858 (2019). 10.1111/jch.13499 PMC8030427 30803128

[65] Duncker, D. et al. Smart wearables for cardiac monitoring—real-world use beyond atrial fibrillation. *Sensors* **21**, 2539 (2021). 33916371 10.3390/s21072539 PMC8038592

[66] Gill, S. K. et al. Consumer wearable devices for evaluation of heart rate control using digoxin versus beta-blockers: the RATE-AF randomized trial. *Nat. Med.* **30**, 2030–2036 (2024). 39009776 10.1038/s41591-024-03094-4 PMC11271403

[67] Hernández, C. R. et al. Validation of the portable air-smart spirometer. *PLOS ONE* **13**, e0192789 (2018). 29474502 10.1371/journal.pone.0192789 PMC5825056

[68] Zhou, P., Yang, L. & Huang, Y.-X. A smart phone based handheld wireless spirometer with functions and precision comparable to laboratory spirometers. *Sensors* **19**, 2487 (2019). 31159155 10.3390/s19112487 PMC6603793

[69] Rodbard, H. W. et al. The effect of canagliflozin, a sodium glucose cotransporter 2 inhibitor, on glycemic end points assessed by continuous glucose monitoring and patient-reported outcomes among people with type 1 diabetes. *Diabetes Care* **40**, 171–180 (2016). 27899497 10.2337/dc16-1353

[70] Lawton, M. et al. Parkinson’s disease subtypes in the Oxford Parkinson disease centre (OPDC) discovery cohort. *J. Park. Dis.* **5**, 269–279 (2015). 10.3233/JPD-140523 PMC4923737 26405788

[71] Bloem, B. R. et al. The personalized Parkinson project: examining disease progression through broad biomarkers in early Parkinson’s disease. *BMC Neurol.* **19**, 160 (2019). 31315608 10.1186/s12883-019-1394-3 PMC6636112

[72] Sturchio, A. et al. Phenotype-agnostic molecular subtyping of neurodegenerative disorders: the Cincinnati cohort biomarker program (CCBP). *Front. Aging Neurosci.* **12**, 553635 (2020). 33132895 10.3389/fnagi.2020.553635 PMC7578373

[73] Trebeschi, S. et al. Predicting response to cancer immunotherapy using noninvasive radiomic biomarkers. *Ann. Oncol.* **30**, 998–1004 (2019). 30895304 10.1093/annonc/mdz108 PMC6594459

[74] Johannet, P. et al. Using machine learning algorithms to predict immunotherapy response in patients with advanced melanoma. *Clin. Cancer Res.* **27**, 131–140 (2021). 33208341 10.1158/1078-0432.CCR-20-2415 PMC7785656

[75] Kong, J. et al. Network-based machine learning approach to predict immunotherapy response in cancer patients. *Nat. Commun.* **13**, 3703 (2022). 35764641 10.1038/s41467-022-31535-6 PMC9240063

[76] Noh, B. et al. XGBoost based machine learning approach to predict the risk of fall in older adults using gait outcomes. *Sci. Rep.* **11**, 12183 (2021). 34108595 10.1038/s41598-021-91797-w PMC8190134

[77] Connie, T. & Zhe Khae, L. Fall risk prediction using temporal gait features and machine learning approaches. *Front. Artif. Intell*. **7**, 1425713 (2024). 10.3389/frai.2024.1425713 PMC11389313 39263525

[78] Bonkhoff, A. K. & Grefkes, C. Precision medicine in stroke: towards personalized outcome predictions using artificial intelligence. *Brain* **145**, 457–475 (2022). 34918041 10.1093/brain/awab439 PMC9014757

[79] Mainali, S., Darsie, M. E. & Smetana, K. S. Machine learning in action: stroke diagnosis and outcome prediction. *Front. Neurol*. **12**, 734345 (2021). 10.3389/fneur.2021.734345 PMC8685212 34938254

[80] Cheon, S., Kim, J. & Lim, J. The use of deep learning to predict stroke patient mortality. *Int. J. Environ. Res. Public Health* **16**, 1876 (2019). 31141892 10.3390/ijerph16111876 PMC6603534

[81] Castelletti, S. et al. A wearable remote monitoring system for the identification of subjects with a prolonged QT interval or at risk for drug-induced long QT syndrome. *Int. J. Cardiol.* **266**, 89–94 (2018). 29887480 10.1016/j.ijcard.2018.03.097

[82] Tsuji, H. et al. Impact of reduced heart rate variability on risk for cardiac events. *Circulation* **94**, 2850–2855 (1996). 8941112 10.1161/01.cir.94.11.2850

[83] Thayer, J. F., Yamamoto, S. S. & Brosschot, J. F. The relationship of autonomic imbalance, heart rate variability and cardiovascular disease risk factors. *Int. J. Cardiol.* **141**, 122–131 (2010). 19910061 10.1016/j.ijcard.2009.09.543

[84] Huikuri, H. V. & Stein, P. K. Heart rate variability in risk stratification of cardiac patients. *Prog. Cardiovasc. Dis.* **56**, 153–159 (2013). 24215747 10.1016/j.pcad.2013.07.003

[85] Zheng, N. S. et al. Sleep patterns and risk of chronic disease as measured by long-term monitoring with commercial wearable devices in the All of Us Research Program. *Nat. Med.* **30**, 2648–2656 (2024). 39030265 10.1038/s41591-024-03155-8 PMC11405268

[86] Zhang, J. et al. Association of sleep duration and risk of mental disorder: a systematic review and meta-analysis. *Sleep Breath* **28**, 261–280 (2024). 37642884 10.1007/s11325-023-02905-1 PMC10954977

