# Supplementary material for: Challenges and potential of using digital biomarkers in healthcare and clinical trials
Source: Commun Med (Lond). 2026 Feb 21;6:151. doi: 10.1038/s43856-026-01450-8 (PMC12996552; doi:10.1038/s43856-026-01450-8)
Supplement: Supplementary file 1 — Description of Additional Supplementary Files [file 43856_2026_1450_MOESM1_ESM.docx]

Description of Additional Supplementary Files

Supplementary Data 1: A comprehensive version of Table 1, providing complete definitions and examples of traditional and digital biomarkers
